# Dengue virus 4/2 envelope domain chimeric virus panel maps type-specific responses against dengue serotype 2

**DOI:** 10.1128/mbio.00818-23

**Published:** 2023-10-06

**Authors:** Deanna R. Zhu, Alecia J. Rajesh, Rita M. Meganck, Ellen F. Young, Jennifer E. Munt, Victor L. Tse, Boyd Yount, Helen Conrad, Laura White, Sandra Henein, Aravinda M. DeSilva, Ralph S. Baric

**Affiliations:** 1 Department of Epidemiology, Gillings School of Global Public Health, University of North Carolina at Chapel Hill, Chapel Hill, North Carolina, USA; 2 Department of Molecular Microbiology and Immunology, School of Medicine, Saint Louis University, St. Louis, Missouri, USA; 3 College of Science, Engineering and Food Science, University College Cork, Cork, Ireland; 4 Department of Microbiology and Immunology, School of Medicine, University of North Carolina at Chapel Hill, Chapel Hill, North Carolina, USA; Johns Hopkins Bloomberg School of Public Health, Baltimore, Maryland, USA; University of Texas Medical Branch, Galveston, Texas, USA

**Keywords:** dengue, neutralizing antibodies, reverse genetics

## Abstract

The DENV envelope (E) and pre-membrane (prM) glycoproteins are primary targets of serologic immunity after infection and vaccination. Of these, serotype-specific (TS) antibodies typically target E domains, while serotype cross-reactive (CR) antibodies typically the target prM protein and conserved E regions. To identify and quantify E-domain TS neutralizing antibody responses in polyclonal sera, we developed a panel of chimeric DENV4/2 viruses that incorporate DENV2 envelope domain I, II, and III (DENV4/2-EDI, EDII, EDIII) into the DENV4 E glycoprotein. Chimeric DENV4/2 viruses were recovered, replicated efficiently, and displayed similar maturation states as parental viruses. The recovery of viable DENV4/2-EDII recombinants required the inclusion of chimeric DENV4/2 prM that maintained critical interactions with chimeric E. To assess structural integrity and epitope display of chimeric viruses, we examined neutralization of mature virions by monoclonal antibodies (mAbs) and heterotypic polyclonal sera. The ED-chimeric virions preserved epitopes of TS and envelope-dimer-epitope CR mAbs and had similar sensitivity to CR polyclonal responses as parental strains. Primary sera from natural infection and human challenge target a region centered on EDIII and secondarily target EDII and EDI. Sera from natural infection had a unique neutralization pattern compared to sera from human challenge, which included greater frequency and higher titer of responses against DENV EDII. In summary, DENV4/2 E recombinant viruses delineate the subdomain targets of TS antibodies after vaccination and primary infection, which may provide new correlates of protection or identify epitopes of neutralizing monoclonal antibodies.

**IMPORTANCE:**

The four dengue virus (DENV) serotypes infect several hundred million people each year. Although primary infection is generally mild, subsequent infection by differing serotypes increases the risk for symptomatic disease ranging from fever to life-threatening shock. Despite the availability of licensed vaccines, a comprehensive understanding of antibodies that target the viral envelope protein and protect from infection remains incomplete. In this manuscript, we develop a panel of recombinant viruses that graft each envelope domain of DENV2 onto the DENV4 envelope glycoprotein, revealing protein interactions important for virus viability. Furthermore, we map neutralizing antibody responses after primary DENV2 natural infection and a human challenge model to distinct domains on the viral envelope protein. The panel of recombinant viruses provides a new tool for dissecting the E domain-specific targeting of protective antibody responses, informing future DENV vaccine design.

## INTRODUCTION

The global incidence of disease caused by the four dengue virus (DENV) serotypes has increased 15-fold in the last two decades (1). This trend is predicted to continue as environmental and ecological changes favor the survival and spread of DENV mosquito vectors (2, 3). Currently, half of the global population lives in areas that place them at risk of infection by one or more DENV serotypes, of which dengue serotype 2 (DENV2) has the greatest incidence. Although vaccination has great potential to prevent and eliminate arthropod-borne viral diseases, antibodies elicited against one dengue serotype can enhance disease caused by a different serotype, termed antibody-dependent enhancement (ADE). Furthermore, protection from disease is not solely defined by the presence of neutralizing antibodies but also their epitope targets and effector functions (4). After a primary infection both serotype-specific [type-specific (TS)] and serotype-cross-reactive (CR) antibodies are induced, but TS responses best correlated with protection (5). TS responses have been mapped to specific epitopes on the viral E, but the polyclonal serologic repertoire of E domain targets recognized by neutralizing antibodies remains uncertain.

The DENV envelope glycoprotein, comprised of surfaced-exposed envelope domains I, II, and III (EDI, EDII, and EDIII), is the main target of neutralizing antibodies after infection or vaccination. The E glycoprotein is responsible for viral attachment and entry into host cells and, in the mature virion, presents as 30 rafts each made of three homodimers arranged in a herringbone pattern (6). After viral entry, endosome acidification induces conformational changes of the E in which EDII lifts away from the viral surface as EDIII rotates down; three EDII form the arm of one heterotrimer in the fusion conformation (7, 8). In infected cells, new DENVs are assembled within the endoplasmic reticulum as immature virions containing 60 surface E and pre-membrane (prM) protein heterotrimers in a spiked form (9). During viral egress through the secretory pathway, the cellular protease furin cleaves prM, which primes the reorganization of E proteins into the smooth, mature infectious virus at neutral pH (10). When DENV is produced on standard laboratory cells lines, prM processing is incomplete, and new virions released from the cells contain a mixture of immature, partially mature, and fully mature viruses (11). While the maturation state of DENVs circulating in humans has not been comprehensively evaluated across serotypes and genotypes, one study using viremic blood samples derived from a DENV1 patient during an epidemic in Sri Lanka demonstrated that virions in human plasma are more infectious and mature than laboratory grown virions (12). The DENV maturation state influences structural and functional properties of individual E proteins on the viral envelope (13). Compared to fully mature viruses, immature and partially mature viruses are more sensitive to neutralization by low-affinity, serotype cross-reactive antibodies (12).

After primary DENV exposure or vaccination, TS and CR antibodies are elicited that target the E and prM glycoproteins. In general, CR antibodies wane after a few years in most individuals and are associated with increased risk for ADE, while TS neutralizing antibodies are long-lasting and associated with protection. After a secondary infection, neutralizing antibodies have been found to target complex epitopes across the envelope dimer epitope (EDE) or the bc region of EDII beside the fusion loop on multiple serotypes, which have potent CR neutralizing ability (14, 15).

Using functional epitope mapping with chimeric DENV3 virions, we previously identified 15 TS neutralizing antibodies isolated from individuals after secondary infection who had experienced either primary or secondary DENV3 infection, which targeted six to seven distinct antigenic sites localized in each envelope domain (ED) of DENV3 (16). It remains uncertain whether other DENV serotypes elicit focused or broad ED responses after infection or vaccination. Previously, an assessment of DENV2 TS neutralization antibodies showed that human monoclonal antibodies (mAbs) 2D22 and 1L12 target two distinct but overlapping quaternary epitopes that span EDIII and EDII encoded by different monomers and that antibody 3F9 that targets EDI (17). Assessment of DENV2 natural infection and human challenge sera showed that most individuals targeted either or both EDIII and EDI (17), yet specific targets in E subdomains in polyclonal serum and the maturation dependence of neutralization remain to be determined.

**Fig 1:**
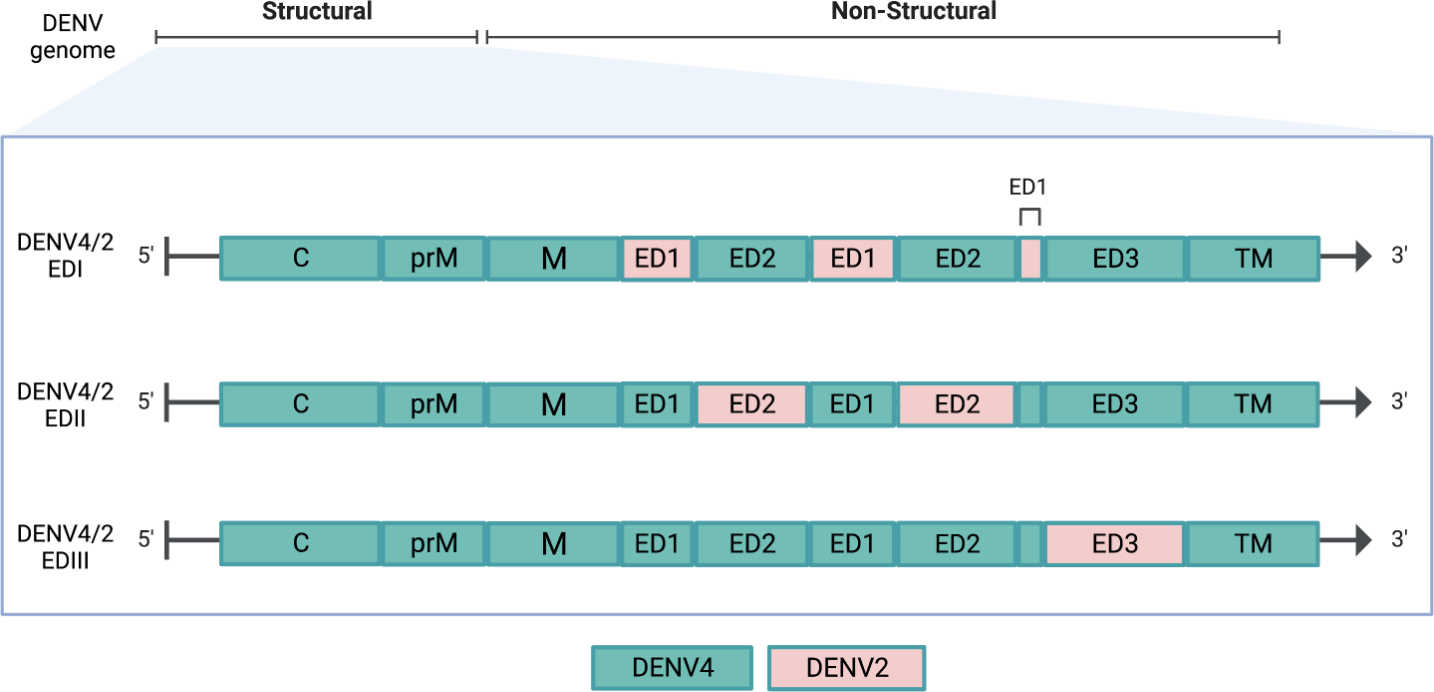
The three chimeric DENV4/2 ED chimeric viruses have each surface-exposed ED of DENV4 replaced with DENV2 residues. (**A**) Organization of DENV genome and schema of changes of the chimeric panel. DENV2 regions are shown in pink, and DENV4 regions are in blue-green. C, capsid; prM, pre-membrane; M, membrane; ED, envelope domain; TM, transmembrane.

We describe the recovery and characteristics of DENV4 and DENV2 EDI, EDII, and EDIII chimeric viruses that encode each surface-exposed ED of DENV2 S16803 on the DENV4 Sri Lanka ’92 E glycoprotein backbone (Fig. 1). We show that the panel displays authentic, complex neutralizing DENV2 epitopes which are appropriately recognized by domain-specific mAbs. Using the chimeric virus panel, we demonstrate that humans exposed to DENV2 infection have serum antibodies that mainly target EDIII and less frequently EDII or EDI. Demonstrating the ability to measure ED-specific neutralizing titers in polyclonal serum, the DENV4/2 ED chimeric panel can be used to characterize TS antibody responses after vaccination or natural infection in epidemiologic cohorts and capture domain-specific protective responses against DENV.

## RESULTS

### Recombinant DENV4/2 virus design

**Fig 2:**
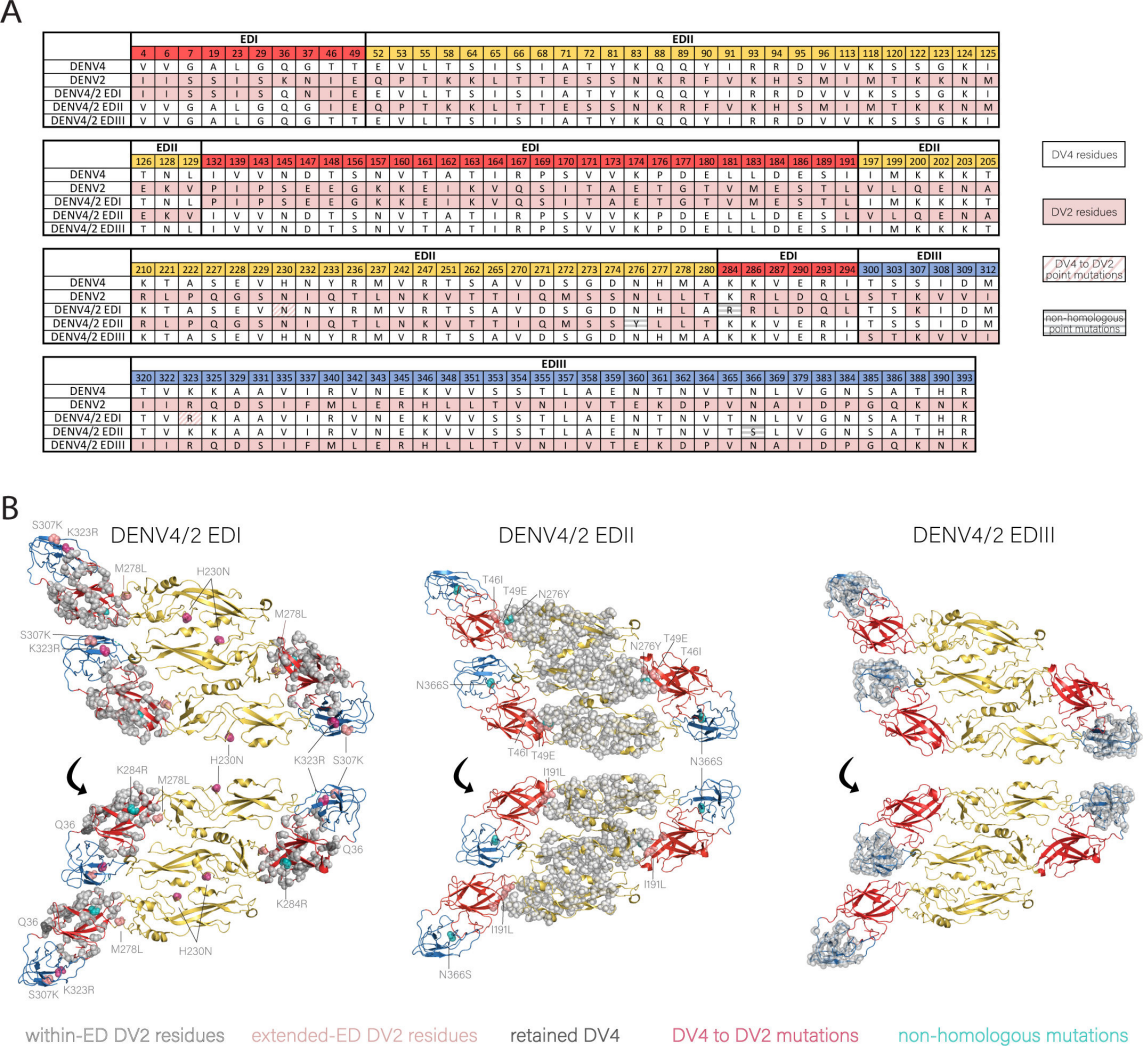
Recovered DENV4/2 ED chimeras display ED epitopes of DENV2. The ED regions are colored in red, yellow, and blue for EDI, EDII, and EDIII, respectively. (**A**) Alignment of all differing amino acid residues on the surface ED between DENV4 and DENV2. Residues designed to be DENV2 are colored pink, while residues kept as DENV4 are white. This includes the additional DENV2 to DENV4 changes made for viral stability (DENV4/2-EDI M278L, S307K), and any residues within the ED that were kept DENV4 (DENV4/2-EDI Q36; DENV4/2-EDII I46, **E49**). Mutations that arose during passaging and then introduced into the molecular clone and rederived (DENV4/2-EDI H230N, K284R, K323R; DENV4/2-EDII N276Y and N366S) are hatched; DENV2 residues are in pink, and residues that are not shared by DENV4 and DENV2 are in gray. (**B**) Three monomers of each DENV4/2 ED chimera with changed residue backbones highlighted (PDB 3J27).

**Fig 3:**
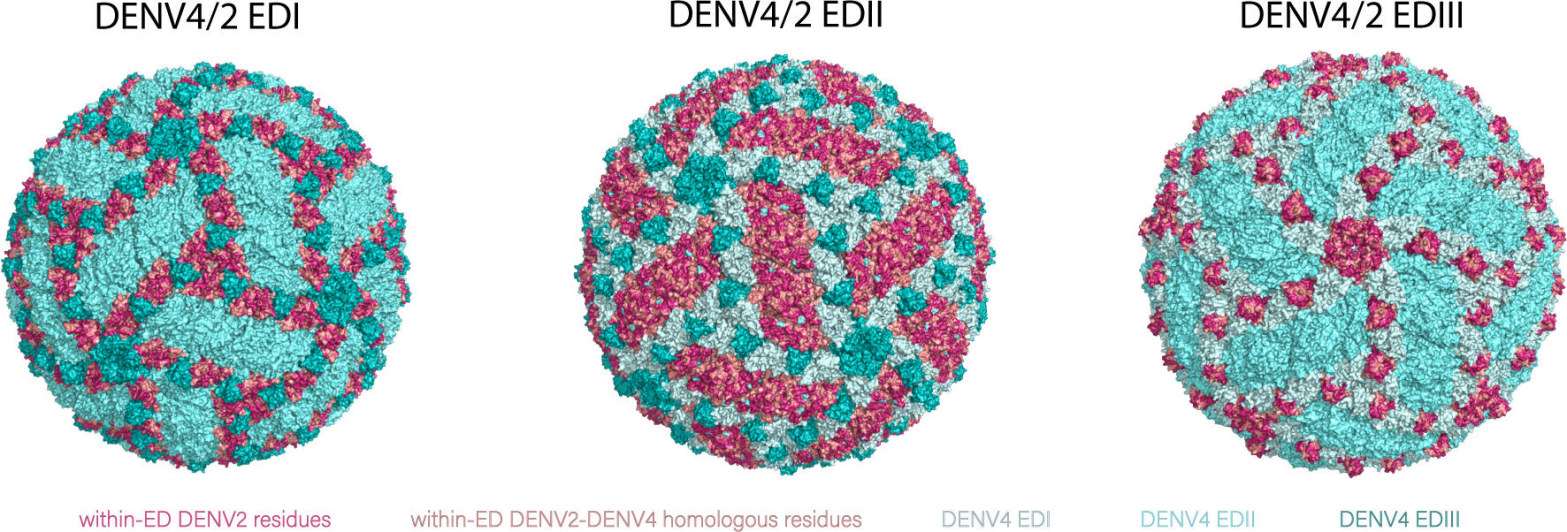
Surface projection of each DENV4/2 ED chimera (PDB 3J27) of DENV4/2-EDI, DENV4/2-EDII, and DENV4/2-EDIII shows preservation of threefold, twofold, and fivefold axes of symmetry.

By evaluating the DENV4 and DENV2 EDI amino acid sequence homology, the chimeric DENV4/2-EDI was designed to include 42 amino acids encompassing the DENV2 EDI (Fig. 2A). Using a structure-guided approach, which involved evaluating published E crystal structures to evaluate the stability of the proposed residues changes, two other residue changes, EDII M278L and EDIII K307S, were included to preserve interactions with EDI residues T49E in the EDI/EDII hinge region of the monomer and R167Q of the neighboring dimer, respectively (Fig. 2A and B). One residue on the intramembrane side of EDI, Q36, was preserved from DENV4 due to interaction with transmembrane residues. Prior recombinant envelope domain chimeras and structural analyses of the dengue virus prM showed that a viable EDII chimera requires the maintenance of homotypic interactions between DENV2 prM and EDII (18). Consequently, the DENV4 glycoprotein backbone was modified by introducing 29 prM and 61 EDII heterologous DENV2 residues, generating the DENV4/2-EDII chimeric virus (Fig. S1; Fig. 2). Again, using a structure-guided approach, residues T46I and T49E were changed from DENV4 to DENV2 on the EDI D_0_ β-sheet to preserve DENV2-DENV2 EDI-EDII interactions within a monomer and interactions with DENV2 hinge residues 277–280 in the fusion structure. The DENV4/2-EDIII virus was generated by introducing 40 DENV2 amino acid changes into DENV4 EDIII. No additional stabilizing point mutations were necessary (Fig. 2). Overall, the recovered chimeras DENV4/2-EDI, DENV4/2-EDII, and DENV4/2-EDIII recombinant viruses presented the DENV2 threefold, twofold, or fivefold axes, respectively, on the surface-exposed E protein (Fig. 3).

### DENV4/2 ED chimera recovery

We utilized a four-component DENV reverse genetics system that has been described previously to generate recombinant chimeric viruses (19, 20). Briefly, the full-length DENV genome was sub-cloned as four contiguous pieces flanked by type IIS restriction endonucleases. Fragments, after restriction digestion and gel purification, were ligated into full-length templates for *in vitro* transcription of genome length RNAs and electroporated into cells. After passaging in Vero 81 cells, the DENV4/2-EDI chimera evolved three point mutations in E (H230N, K284R, and K323R), which were then introduced into the molecular clone and re-derived (Fig. 2). The DENV4/2-EDII virus also evolved tissue culture adaptations in E at positions N276Y and N366S, which were also introduced into the molecular clone and re-derived. No novel mutations were detected in the DENV4/2-EDIII virus (Fig. 2).

### DENV4/2 ED panel characteristics

**Fig 4:**
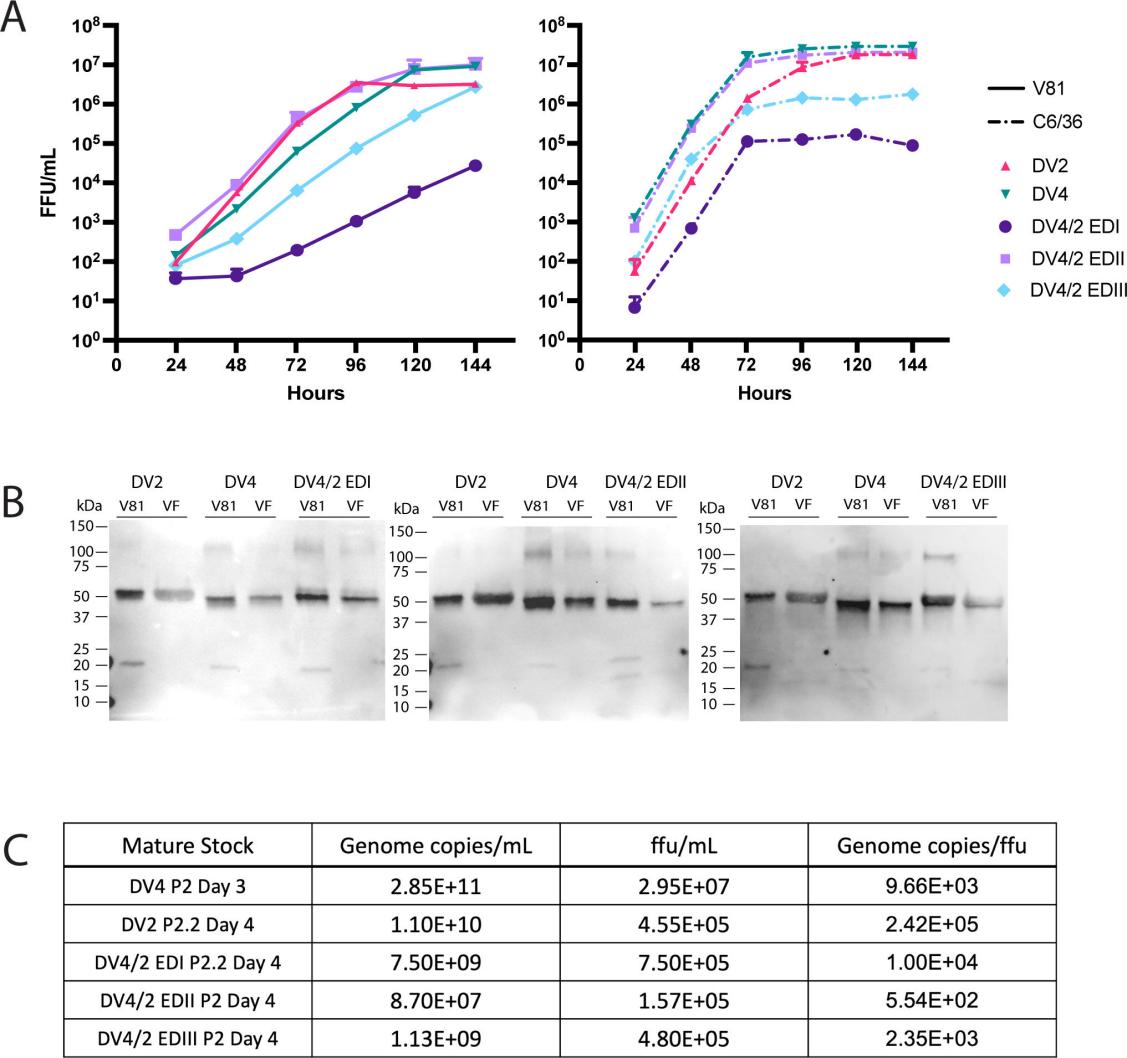
Characteristics of DENV4/2 ED panel and parental strains. (**A**) Growth kinetics of ED panel on Vero 81 (mammalian) and C6/36 (insect) cells at multiplicity of infection of 0.001 in triplicate. DENV4/2-EDI had significantly lower kinetics compared to other viruses on both Vero 81 and C6/36; *P*s < 0.05. All viruses had significantly different growth kinetics on Vero 81 and C6/36 cells, *P*s < 0.05. (**B**) Western blot of virions against envelope (53 kDa) and prM (21 kDa) proteins from stocks grown on Vero 81 and Vero furin-overexpressing (VF) cells, conducted in triplicate. Each representative image consists of Vero 81 and VF stocks of parental strains DENV2 (lanes 1–2), DENV4 (lanes 3–4), and each ED chimera (lanes 5–6), from left to right, DENV4/2-EDI, EDII, and EDIII. (**C**) Average genome copy number per focus-forming unit (ffu) of mature stocks with RNAse treatment, conducted in duplicate.

In Vero 81 cells, the chimeric viruses form distinct foci morphology, as DENV4/2-EDI and EDIII formed small foci of similar diameter to DENV2 while DENV4/2-EDII formed large, dispersed foci more reflective of DENV4 (Fig. S2). Although DENV2 formed distinctively lighter and more dispersed foci compared to DENV4 in C6/36 cells, the chimeric viruses formed large foci like DENV4. While DENV2, DENV4, DENV4/2-EDII, and DENV4/2-EDIII replicated to similar peak titers in Vero 81 cells, DENV4/2-EDI showed a significant >2 log reduced growth phenotype (Fig. 4A) (*P* < 0.05). DENV4/2-EDI also replicated to lower titers than other viruses on C6/36 cells but displayed similar exponential growth in the first 72 h of infection (*P* < 0.05). All viruses had greater growth kinetics on C6/36 cells compared to Vero 81 cells (*P*s < 0.05).

To control for maturation status in downstream assays, virus stocks were established in furin-overexpressing Vero 81 cells (VF), which produced fully mature virions as previously reported (Fig. 4B) (21). Vero 81 and VF-grown stocks of the parental strains and each chimeric recombinant virus were run in non-reducing SDS-PAGE and probed by anti-E and anti-prM antibodies. Maturation state was assessed by comparing the ratio of E and prM band intensity by Western blot, where lighter or absent prM bands in the presence of E reflected increased maturity in stocks grown in VF cells.

DENV2 had the highest ratio of viral genome copies to focus-forming units (FFU), while DENV4 had ~1 log lower ratio, reflecting greater packaging efficiency. The chimeric viruses had genome to particle ratios similar to the backbone DENV4 strain (Fig. 4C). We observed that the mature, VF-grown viruses were slightly more efficient than their partially mature counterparts (Table S1).

### Neutralization by monoclonal antibodies

**Fig 5:**
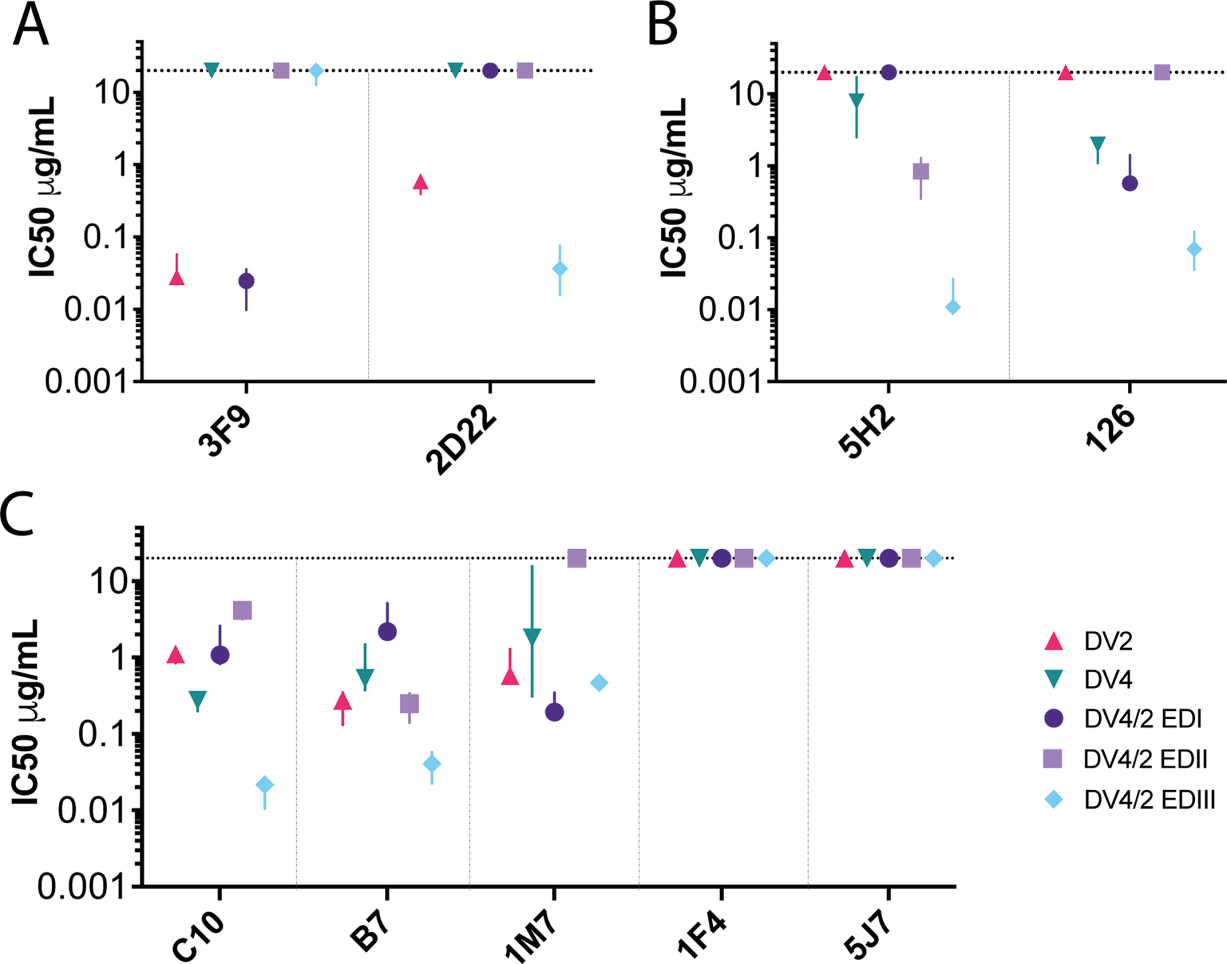
Neutralization of mature chimeric DENV4/2 chimeric viruses by distinct monoclonal antibodies. (**A**) DENV2 (3F9 and 2D22); (B) DENV4 (5H2 and 126); (**C**) cross-reactive, (C10, B7, and 1M7), DENV1 (1F4), and DENV3 (5J7) antibodies. Shapes indicate the median of three to five biological replicates, and lines represent the interquartile ratio.

To assess the structure and preservation of relevant DENV2 and DENV4 TS epitopes on the chimeras, we performed neutralization assays with a panel of well-characterized DENV2 and DENV4 mAbs (Fig. 5). The human mAb 3F9, which primarily binds to DENV2 EDI (13), neutralized the wild-type DENV2 and chimeric DENV4/2-EDI at similar concentrations (Fig. 5A) and did not neutralize DENV4/2-EDII, DENV4/2-EDIII, or DENV4. Similarly, the human neutralizing mAb 2D22, which binds to conserved residues on DENV2 EDII and variable residues in EDIII on different monomers (22, 23), neutralized the wild-type DENV2 and chimeric DENV4/2-EDIII but not DENV4, DENV4/2-EDI, or DENV4/2-EDII viruses (Fig. 5A). The neutralization titers indicated that the DENV4/2-EDI chimera maintained the EDI epitope targeted by 3F9 and that the critical epitope residues involved in serotype-specific DENV2 neutralization by 2D22 were captured by the chimeric DENV4/2-EDIII virus but not the DENV4/2-EDI or EDII virus.

The non-human primate mAb 5H2, which targets the DENV4 EDI (24), neutralized DENV4, DENV4/2-EDII, and DENV4/2-EDIII, but not DENV4/2-EDI (Fig. 5B). Human mAb 126, which targets an epitope in the DENV4 EDI/EDII hinge region (25) neutralized DENV4, DENV4/2-EDI, and DENV4/2-EDIII, but not DENV4/2-EDII or DENV2.

All mature parental and ED chimeric viruses were efficiently neutralized by the dimer dependent, cross-neutralizing antibodies C10 and B7 (14) (Fig. 5C). All viruses except the mature DENV4/2-EDII chimera were neutralized by the cross-reactive antibody to the fusion loop, 1M7. FRNT of mature and immature viruses showed that mature stocks were more resistant to neutralization by 1M7 than immature stocks, consistent with earlier studies (Fig. S3) (21). None of the viruses were neutralized by antibodies specific to DENV1 (1F4) or DENV3 (5J7) (Fig. 5C) (26, 27). Together, the ED panel is sensitive to neutralization by the appropriate domain-specific mAbs from DENV2 and DENV4 and are not neutralized when the appropriate ED is missing from the recombinant viruses.

### Neutralization by heterotypic convalescent sera

**Fig 6:**
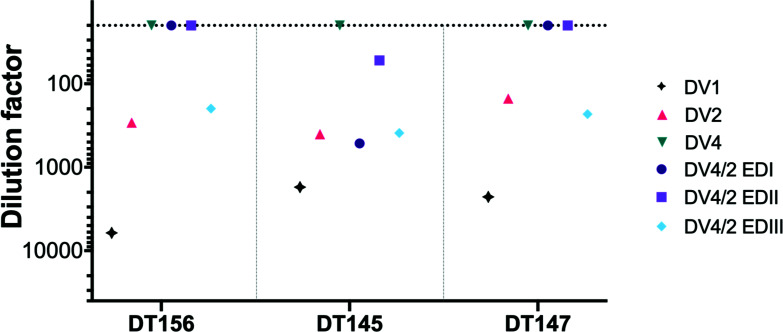
Neutralization of DENV4/2 ED panel by heterotypic DENV1 convalescent sera reflects neutralization sensitivity of parental DENV2. FRNT was conducted on Vero 81 cells in duplicate and incubated for 45–48 h at 37°C.

Next, we assessed the viral panel with polyclonal sera from individuals with known infection histories (Table S2). To develop the chimeric viruses for measurement of TS antibody responses, neutralization by heterotypic convalescent sera was used to evaluate the chimeric virus sensitivity against cross-reactive sera compared to the parental strains (Fig. 6). Primary DENV infections often elicit CR weakly neutralizing antibodies targeting other serotypes, often toward the prM or fusion loop epitope (28 – 31). In addition, subcomplex-specific antibodies, like mAb 1A1D-2, recognize an epitope in EDIII and neutralizes DENV serotypes 1–3, but not DENV4 (32, 33).

Convalescent DENV1 primary natural infection sera neutralized DENV1 5-fold to 20-fold more efficiently than DENV2, while no neutralization was observed for DENV4 (Fig. 6). DENV4/2-EDIII was neutralized by all samples at a similar dilution as DENV2, suggesting that DENV2 EDIII was a primary target for CR antibody. One sample (DT145) contained more complex combinations of CR antibodies as sera neutralized all DENV4/2 chimeras. As a tool to identify DENV2 TS antibodies from polyclonal responses, the chimeras are no more sensitive to heterotypic neutralization than the parental DENV2 virus. Furthermore, the data suggest that measures of TS antibody responses in primary DENV1 sera may include low-abundance CR antibodies.

### Neutralization by DENV2 convalescent sera

To evaluate the ED targets of homotypic neutralizing antibodies in convalescent sera, DENV2 serum samples were obtained from a traveler’s cohort and human challenge study (34, 35) (Table S2). To demonstrate that the reagent panel can separate domain-specific targets of a polyclonal antibody response, a high-titer DENV2 convalescent sample (110) and two moderate-titer samples (1,737 and 1,996) were depleted by three conditions: a control depletion of bovine serum albumin (BSA); a combination of DENV1, DENV3, and DENV4 (heterotypic depletion); and a combination of DENV2 and DENV4 (homotypic depletion) (Fig. S4). For the high-titer sample 110 with neutralization against the DENV4 backbone virus, heterotypic depletion removed neutralization against DENV4/2-EDI, suggesting that antibodies targeting EDI were cross-reactive. Assessment of the type-specific response, calculated as the difference in IC_50_ between the heterotypic and homotypic depletions for each virus, was directed towards DENV4/2-EDII and DENV4/2-EDIII (Fig. S4A; Table S3). For the two moderate-titer samples without DENV4 neutralization, heterotypic depletion did not alter the neutralization phenotype, indicating that TS responses were responsible for neutralization. The type-specific response against DENV2 for 1737 showed similar targeting to DENV4/2-EDII and DENV4/2-EDIII, while 1996 only targeted DENV4/2-EDIII (Figure S4B and C; Table S3).

**Fig 7:**
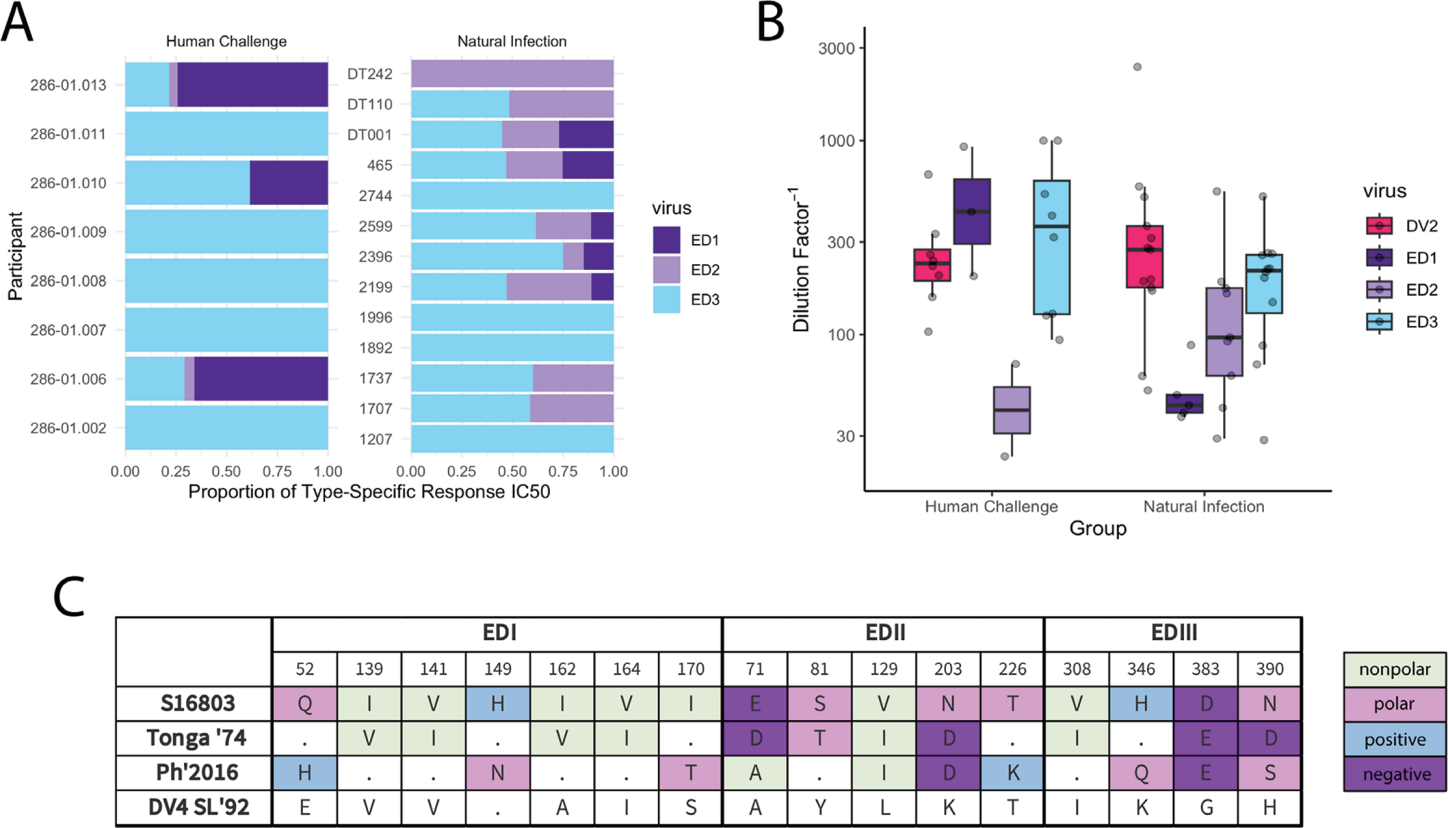
Neutralization of DENV4/2 chimeric viruses by primary DENV2 sera after natural infection and human challenge. (**A**) Proportion of type-specific responses to each envelope domain of DENV2 in primary sera after human challenge and natural infection. (**B**) Boxplot and overlaid scatterplot of DENV2, DENV4/2-EDI, DENV4/2-EDII, and DENV4/2-EDIII IC_50s_ in human challenge and natural infection cohorts. (**C**) Non-homologous residues between DENV2 parental strain S16803, human challenge strain Tonga ’74, and a contemporary strain from Southeast Asia that is relevant for the majority (10/13) of the natural infection cohort. Residues from the DENV4 parental strain are provided as a reference. Dots represent homologous residues to S16803.

We then evaluated additional DENV2 convalescent samples to obtain a total of 13 samples from natural infection cohorts and 8 samples after human challenge (34, 35) (Table S2). As none of these samples neutralized the backbone virus DENV4, no depletion was required to remove CR antibodies for evaluation of TS responses (Fig. 7; Fig S5A and B). Considering the overall proportion of the TS response against EDI, EDII, and EDIII, the majority of TS responses targeted EDIII followed by EDII, then EDI (Fig. 7A). The neutralization of DENV2 did not significantly differ across the two groups, and this was reflected in EDIII neutralization (Fig. 7B) (W = 39, *P* = 0.37 and W = 51, *P* = 0.97, respectively). Those that experienced natural infection targeted EDII more frequently than those that received a DENV2 challenge (25% vs 69.2%) and at significantly greater titers (W = 23, *P* = 0.03). While convalescent sera from natural infection targeted EDI at a similar frequency to that of human challenge studies (37.5% vs 38.4%), they had lower titers that did not reach statistical significance (Fig. 7B; Table S5) (W = 45, *P* = 0.61).

We then evaluated whether the differences in targeting against EDI and EDII were related to genotypic differences between the human challenge Tonga ’74 strain (X54319) and a representative strain of circulating DENV2 in our natural infection cohort (MG895171) (Fig. 7C; Fig. S6). The human challenge strain had no charge differences in the residues that differed on EDI, while a circulating DENV2 strain had three residue differences with charge differences (polar to positive, positive to polar, and nonpolar to polar). On EDII, Tonga ’74 differed on four residues, with one charge change from polar to negatively charged, while the circulating strain had four mismatched residues and three charge differences (negative to nonpolar, polar to negative, and polar to positive). The higher titers of EDI neutralization in the human challenge samples may be attributable to the relatively little charge differences between the infecting strain and the chimeric EDI virus. On the other hand, the charge differences on EDII are not as clearly correlated with the observed differences in the neutralizing ability of the sera measured by the IC_50_ between the two groups.

## DISCUSSION

DENV vaccine development has been hampered by uncertain correlates of protective immunity, exemplified by the occurrence of breakthrough infections in individuals with relatively high levels of neutralizing antibodies and T-cell responses (36). One licensed DENV vaccine, Dengvaxia, is not recommended for use in naïve populations due to an increased risk of severe DENV after vaccination, and another, the TAK003 tetravalent vaccine, has been recently licensed in Indonesia and the European Union for use in individuals age 4 and older (37). The possibility of nuanced antibody responses in the setting of new and continued vaccination efforts supports the need for improved correlates of protective immunity (38). For DENV2, prior studies have identified an important antigenic site in EDI and a major site that spans EDII-EDIII, yet the totality of the ED TS neutralizing antibody pools remains uncertain in serum after natural infection and vaccine sera (17, 23).

We designed, recovered, and characterized a panel of DENV4/DENV2 E-domain chimeric recombinant viruses that encode DENV2 EDI, EDII, and EDIII sequences in the DENV4 E glycoprotein backbone. The E glycoprotein of DENV4, the serotype with the lowest global annual incidence, is genetically and antigenically distant from DENV2 E (39), providing a suitable backbone to evaluate DENV2 ED type-specific antibody responses in human populations. The DENV4/2 chimeric panel replicates efficiently in mammalian and mosquito cells and produces virions with similar maturation states as parental wild-type viruses.

Building on recent studies by our group, the recovery of replication competent chimeric DENV4/2-EDII recombinant viruses was dependent on a chimeric DENV prM glycoprotein (18). These data support earlier studies that have demonstrated intimate interactions between prM and E are essential for virus viability. The mutations in the chimeric prM likely preserve transmembrane interactions between DENV2 prM and DENV2 EDII as well as DENV4 prM and DENV4 EDIII, which participate in entry, maturation, and release.

It is also apparent that interactions between the three ED are critical for virus replication and are the targets of natural selection. For example, DENV4/2-EDI was less fit, replicating ~2 logs less efficiently in mammalian, but not insect cells. Second-site amino acid changes emerged quickly during passage to improve the replication and recovery of DENV4/2-EDI and DENV4/2-EDII recombinant viruses. These amino acid changes reside at inter-raft, inter-dimer, and within monomer structures that possibly improve stability and presentation of E. For DENV4/2-EDI, the EDII DENV4 to DENV2 H230N change has been previously described using a DENV2/4-EDII chimera where the EDII of DENV4 was placed in the backbone of DENV2, supporting speculation of its potential role in raft-raft stabilization (18). Structural modeling of the DENV2 monomer with K284R and K323R suggests stabilization of the monomer by van der Waal and polar interactions (40). Specifically, K284R may have improved interaction with stem D421, while EDIII K323R is stabilized by the DENV4 to DENV2 EDI T148E change. These assessments can be extended to mutations in the DENV4/2 EDII virus. The N276Y mutation, which resides within the EDI-EDII hinge region, extends toward N128K of EDII, which was altered in the DENV4/2-EDII. Residue N366S is not recognized as a contact interface site in the DENV2 E polyprotein in the mature, pre-fusion form (41) but may help to stabilize the viral post-fusion state, where it interacts with H27 and G28 of EDI, two conserved residues in DENV2 and DENV4 (6). Atomic level resolution of these evolved recombinant viruses should provide a more comprehensive analysis of function and perhaps identify new interaction networks associated with E-prM organization and virion stability.

In agreement with the exchanged E domains, the chimeric recombinant viruses were neutralized by appropriate TS DENV2 and DENV4 mAbs as well as and EDE1 and EDE2 cross-reactive antibodies. The mAb 1M7, a CR antibody that targets fusion loop residues, neutralized all viruses but the mature DENV4/2-EDII. Furthermore, all mature viral stocks were more resistant to neutralization by 1M7 than their partially mature counterparts. This reflects a similar pattern shown by the mAb E53 against West Nile virus (42). The neutralization of each ED chimeric recombinant virus by mAbs indicates that their epitopes are well represented on the surface of each viral E protein.

Some notable variant phenotypes were also evident. The DENV4/2-EDIII virus was more sensitive to neutralization by TS and CR mAbs compared to parental and other ED chimeric viruses but was not evident in polyclonal sera. This may reflect altered particle breathing patterns or a more open virion structure where cryptic or partially occluded epitopes are more exposed and accessible to neutralization. The number and types of interactions between residues across dimers and rafts are thought responsible for openness of the full E structure (43). Out of the 86 electrostatic and 24 hydrogen interraft interactions predicted on a single raft, the DENV4/2-EDIII chimera likely removed eight electrostatic interactions (at residues 309, 346, 384, and 325) and two hydrogen bonds (residue 343). While none of the EDIII changes likely impacted interdimer or E to prM interactions on a raft, they may have altered molecular dynamics, making epitopes more accessible to antibody neutralization. The recombinant panel, coupled with structural studies, may provide a strategy to identify the molecular mechanisms and interaction networks governing the relationship among particle dynamics, antibody occupancy, and neutralization.

Gallichotte et al. used a genetically distinct DENV4/2-EDIII virus to show that DENV2 natural infection and vaccine sera primarily target a neutralizing epitope captured by mAb 2D22 that spans the DENV2 serotype-specific EDIII and conserved EDII residues and, to a lesser extent, EDI in most individuals (17). Here, we generated a complete set of chimeric E-domain recombinant viruses and used them to evaluate E-domain level targets by TS neutralizing antibody responses, controlling for maturation status. Our data support previous findings that the major target against DENV2 lies in a region on EDIII and identifies that the frequency and potency of antibody targets against EDI and EDII may differ with infecting strain or loading dose of an infection. Importantly, the presence of novel antibodies targeting EDII supports the need for new antibody campaigns that map these important epitope domains in DENV2.

In summary, this chimeric panel provides a powerful tool to evaluate E and prM residue interactions that function in virion infectivity, stability, and breathing. Moreover, the panel can be used to identify domain-specific targets of antivirals or monoclonal antibodies. Although highly speculative, the potency and number of ED targeting antibody responses in polyclonal sera may influence the frequency and severity of breakthrough infections. One limitation of the study is that DENV4/2-EDIII chimeras demonstrate increased sensitivity to mAbs; however, this is not noted in the other recombinant viruses. Furthermore, there were limited human challenge and natural infection samples available for robust statistical analyses comparing the two groups. The strengths of this study include the use of furin over-expressing cell lines that readily produce highly mature chimeric viruses similar to wild-type strains and the use of depletion studies that provide additional opportunities to discriminate ED-specific responses in complex serum.

One key finding is that DENV2 primary infections and DENV2 human challenge models elicit variable responses that have predominantly target a region centered on EDIII but have secondary targets on EDII and EDI, potentially influencing neutralizing antibody performance, magnitude, and durability. Similar findings have been reported after DENV3 natural infections (16). Consequently, the DENV4/2 ED panel, when combined with well-designed cohort studies, provides novel opportunities to evaluate whether ED-specific antibody responses correlate with protection after natural infection, vaccination, or breakthrough potential. Future studies that aim to characterize DENV2 ED-level responses in human populations will require greater sample size and control for confounders such as epidemic year and serostatus.

## MATERIALS AND METHODS

### Cells and viruses

Vero 81 cells (ATCC CCL-81) were maintained in DMEM/F12 (Gibco) with 5% fetal bovine serum (FBS; Hyclone), 1× antibiotic-antimycotic (Gibco), 0.1 mM nonessential amino acids (Gibco), 1× GlutaMAX Supplement (Gibco), and 0.075% sodium bicarbonate (Gibco), cultured at 37°C with 5% CO_2_. C6/36 cells (ATCC CRL-1660) were maintained in MEM (Gibco) with 5% FBS (HyClone), 1% penicillin/streptomycin (Gibco), and 0.1 mM nonessential amino acids (Gibco), cultured at 32°C with 5% CO_2_. During virus infection in Vero 81 cells at 37°C, culture media contained 2% FBS (Hyclone), 1% anti-anti (Gibco), 0.1 mM nonessential amino acids (Gibco), 1× GlutaMAX Supplement (Gibco), and 0.075% sodium bicarbonate (Gibco). During virus infection in C6/36 cells, cells were maintained in Opti-MEM (Gibco) with 2% FBS (HyClone), 1% penicillin/streptomycin (Gibco), and 0.1 mM nonessential amino acids (Gibco) at 32°C. Dengue viruses used in this study include DENV1 WestPac 74 (U88535.1), DENV2 S16803 (GU289914.1), DENV3 CH53489 (DQ863638), and DENV4 Sri Lanka 92 (KJ160504.1).

### Dengue reverse genetics system

All viruses were recovered using previously described four-component DENV reverse genetics system (16, 19, 23, 27). Briefly, the full-length DENV genome was sub-cloned as four contiguous pieces flanked by type IIS restriction endonucleases. Plasmids were grown in *E. coli*, purified, and digested with the appropriate restriction endonucleases. Fragments, purified from agarose gels, were ligated overnight with T4 DNA ligase (Thermo Fisher), and full-length RNA was generated by T7 transcription (Thermo Fisher). The genome length RNAs were then electroporated into either C6/36 or Vero 81 cells (Gene Pulser Xcell Bio-Rad). After 4 to 5 days of infection, the supernatants were harvested, passaged, and sequenced.

### Chimeric E recombinant virus design

Amino acid sequences of DENV4 Sri Lanka 92 strain (NCBI KJ160504.1) and DENV2 S16803 (NCBI GU289914.1) were aligned using Geneious. The domain structures of the prM and E glycoproteins of published structures were used to identify residues within each ED. In the DENV4/2-EDI recombinant virus, DENV2 E glycoprotein residues 280–332, 412–475, and 561–578 were introduced into the E glycoprotein of the DENV4 molecular clone. To design the DENV4/2-EDII chimera, DENV2 prM residues 114–190 and E residues 332–412 and 475–561 were synthesized into the DENV4 molecular clone, while the DENV4/2-EDIII recombinant introduced residues from 578 to 680 from DENV2 into the DENV4 backbone (Fig. 1A). The final amino acid sequence of prM and E was then reviewed in Geneious. To evaluate secondary and tertiary structure, the introduced amino acid residues were highlighted on the half-raft and whole virion crystal structure of DENV2 in Pymol (3J27). Several derivatives of each virus were made with variations on residues that could impact E folding and stability.

### Focus-forming assays and focus reduction neutralization assays

Viruses were passaged in cell culture for 3 to 5 days. Focus-forming assays (FFA) and focus reduction neutralization assays (FRNT) were conducted on Vero 81 cells seeded 2E4 cells/well the day prior. The cultures were incubated for 48 h at 37°C. To determine neutralization titers, antibodies, initially concentrated at 33 µg/µL, and sera, starting at a 1:20 or 1:40 dilution, underwent twofold to fourfold serial dilutions. For FRNT, 2.2E3–2.8E3 FFU/mL of virus was mixed with the antibodies or sera in a 1:1 ratio and allowed to incubate at 37°C for 1 h. After culture media were removed from the cells, samples were added onto cells for 1 h at 37°C. After incubation, the cells were overlaid with 1% methylcellulose in Opti-MEM I supplemented with 2% FBS, 0.1 mM nonessential amino acids (Gibco), and 1% penicillin/streptomycin (Gibco) then returned to incubation.

### Immunostaining

After 46–48 h of incubation, the cells were washed with phosphate-buffered saline (PBS) and fixed with 10% buffered formalin. The cells were blocked in 5% milk in permeabilization buffer (eBioscience), incubated at 37°C with cross-reactive antibodies 4G2 and 2H2, washed with PBS, and then incubated at 37°C with HRP-conjugated goat anti-mouse IgG (Sigma). The wells were washed with PBS and then visualized with KPL TrueBlue Peroxidase Substrate (SeraCare). Dry wells were imaged (CTL Analyzer), counted using Viridot on RStudio (version 2022.07.0+548 for macOS), and foci counts were reviewed for accuracy (44).

### Growth curves

Virus growth curves were conducted in six-well format in biological triplicate with multiplicity of infection of 0.001. A 350-µL sample was collected every 24 h and replaced with infection media. Samples from each time point were enumerated as described above.

### Sequencing

For Sanger sequencing, viral RNA was isolated with QIAamp viral RNA kit (Qiagen), and cDNA was generated by SuperScript IV (Thermo) with reverse primers in NS1 (Table S5). Amplicons of E were prepared for sequencing using Q5 Hot Start DNA polymerase (NEB) and forward and reverse primers in C and NS1 (Table S5) and purified using DNA Gel Purification (Qiagen). For deep sequencing, RNA was extracted using Trizol-LS per manufacturer’s specifications (Thermo). Whole-length RNA was prepared using Illumina Stranded Total RNA prep with Ribo-Zero Plus and amplified using Illumina P5 and P7 primers containing 8-nucleotide indexes. Purified PCR products were analyzed on a Bioanalyzer (Agilent Technologies) and quantified on a Qubit 4 fluorometer (Invitrogen). Amplicon libraries were run on a MiSeq system with 2 × 150 bp reads. Results were aligned to the respective viral genome in Geneious Prime.

### Genome copy number to particle forming unit ratio

Viral RNA was diluted and prepared in One-Step RT-ddPCR Advanced kit for probes (Bio-Rad) with primer and probe sets to DENV4 and DENV2 NS5 region (Table S5). Droplets were prepared on the QX200 Automated Droplet Generator, underwent reverse transcription at 50°C and amplification at 59°C × 39 cycles, and read using the QX200 droplet reader. The ratio of genome copy number per milliliter to FFU per milliliter from FFA was compared.

### Viral maturation by Western blot

Virus samples were boiled at 95°C for 5 min in Laemli buffer (Bio-Rad) without reducing reagent. Samples were run on a 4%–20% gradient SDS-PAGE gel at relative volumes to maintain equal ratios of E. Proteins were transferred from gels onto polyvinylidene difluoride membranes by electrophoresis at 100 V for 80 min. The membranes were then blocked with 2% milk in Tris buffered saline and 0.05% Tween. Primary antibodies for human anti-E (1M7) and rabbit anti-prM (Invitrogen PA5-349666) were diluted in blocking buffer and incubated at room temperature for 1 h with rocking or 4°C overnight. The membranes were washed and incubated in goat anti-rabbit HRP (Jackson 111-035-144) and sheep anti-human HRP (GE NA933V) in blocking buffer for 1 h rocking at room temperature. Membrane was washed and visualized with SuperSignal West Pico Plus substrate (Thermo 34577) on iBright FL1500.

### Human serum

All samples used in analysis are summarized in Table S2. Primary DENV1 and DENV2 sera were collected from travelers visiting dengue-endemic countries (UNC IRB protocol 08-0895). Infection history was determined by self-report and FRNT using DENV1-4. DENV2 human challenge study subjects received 10^3^ focus-forming units of the partially attenuated DENV2 human infection virus rDEN2Δ30, Tonga/74 strain, by subcutaneous injection (35). Samples were collected at 180 days after injection (CIR286, Clinicaltrials.gov NCT01931176).

### Whole-virus depletion of antibodies from convalescent sera

Tosylactivated M280 Dynabeads (Invitrogen) were washed three times in 0.1 M borate buffer (pH 9.5) coupled to cross-reactive mAb 1M7 at a ratio of 100-µg 1M7 to 10-mg beads and incubated at 37°C overnight. Beads were washed three times then blocked in PBS with 1% BSA for 30 min at 37°C with end-to-end mixing then buffered in 0.1 M MES, pH 5.6, at 56°C for 30 min and washed in PBS. DENV virions purified by sucrose gradient and BSA controls were conjugated to the coupled beads at a ratio of 100-µg antigen per 5-mg beads with incubation at 37°C for 1 h, then washed three times with PBS pH 7.2. Beads were cross-linked by 2% paraformaldehyde for 20 min at room temperature and then washed four times with PBS pH 7.2. Sera diluted 1:10 in PBS was incubated with conjugated beads for 1 h at 37°C. Supernatant was transferred to new, conjugated beads twice more to complete three rounds of depletion. Depletions were confirmed via enzyme-linked immunosorbent assay as previously described (36, 45, 46).

### Statistical analysis

All comparisons utilized non-parametric tests. Growth curves of log-transformed FFU per milliliter were compared using non-parametric mixed-effect models and Bonferroni correction for multiple comparisons. Neutralization curves were fitted using sigmoidal, 4PL curves against log(concentration in µg/mL) or log(dilution factor) and considered neutralizing when R^2^ >0.75 and Hill Slope >0.5 for sera and R^2^ >0.75 and Hill Slope >0.75 for mAbs. The concentration or dilution factor at which 50% of the virus was neutralized defined IC_50_ and DF_50_. Mann-Whitney tests compared the IC_50_ of each ED-chimeric virus between groups. Analyses were conducted on GraphPad Prism version 9.5.0.

## Data Availability

Consensus sequences for each recombinant virus are available in NCBI under accession no. SRR23784445 to SRR23784447.

## SUPPLEMENTAL MATERIAL

Supplemental Material mbio.00818-23-s0001.pdf Supplemental figures and tables.

ASM does not own the copyrights to Supplemental Material that may be linked to, or accessed through, an article. The authors have granted ASM a non-exclusive, world-wide license to publish the Supplemental Material files. Please contact the corresponding author directly for reuse.
