# Supplementary material for: Dengue virus 4/2 envelope domain chimeric virus panel maps type-specific responses against dengue serotype 2
Source: mBio. 2023 Oct 6;14(5):e00818-23. doi: 10.1128/mbio.00818-23 (PMC10653845; doi:10.1128/mbio.00818-23)
Supplement: Supplemental Material — Supplemental figures and tables. [file mbio.00818-23-s0001.pdf]

	prM																									
	4	7	11	15	16	17	19	21	22	28	29	31	33	37	44	49	52	57	58	59	70	76	82	83	86	89
DENV4	S	D	L	A	K	H	R	R	P	T	E	I	K	I	M	V	K	V	N	T	L	M	Q	S	R	E
DENV2	T	N	H	S	R	Q	K	K	S	E	D	V	M	M	L	I	N	R	Q	N	S	T	T	T	H	E
DENV4/2 EDI	S	D	L	A	K	H	R	R	P	T	E	I	K	I	M	V	K	V	N	T	L	M	Q	S	R	E
DENV4/2 EDII	T	N	H	S	R	Q	K	K	S	E	D	V	M	M	L	I	N	R	Q	N	S	T	T	T	H	G
DENV4/2 EDIII	S	D	L	A	K	H	R	R	P	T	E	I	K	I	M	V	K	V	N	T	L	M	Q	S	R	E

	M																								
	96	99	107	123	125	130	134	135	136	138	139	140	143	146	148	149	152	153	154	156	158	159	160	165	166
DENV4	T	S	A	V	S	N	A	L	L	G	F	M	M	Q	G	I	T	V	F	V	M	M	L	Y	G
DENV2	V	V	T	I	T	H	T	I	M	A	I	L	T	T	H	F	A	L	I	I	L	T	A	M	T
DENV4/2 EDI	T	S	A	V	S	N	A	L	L	G	F	M	M	Q	G	I	T	V	F	V	M	M	L	Y	G
DENV4/2 EDII	V	V	T	V	S	N	A	L	L	G	F	M	M	Q	G	I	T	V	F	V	M	M	L	Y	G
DENV4/2 EDIII	T	S	A	V	S	N	A	L	L	G	F	M	M	Q	G	I	T	V	F	V	M	M	L	Y	G

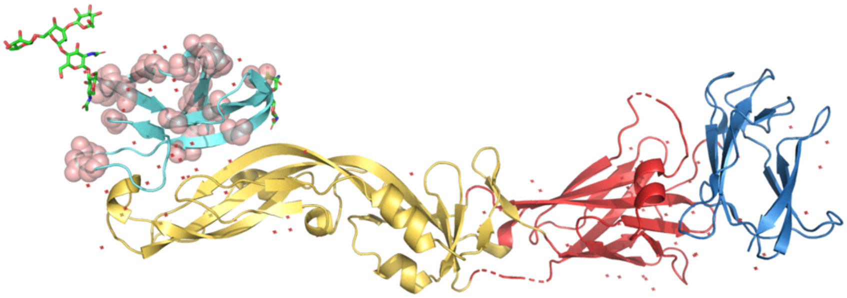

Supplemental Figure 1. DENV4/2-EDII includes a chimeric prM. Residues 1-107 of prM were changed to DENV2. Residues 108-192 remained DENV4. Residue E89G was not shared by DENV4 and DENV2.

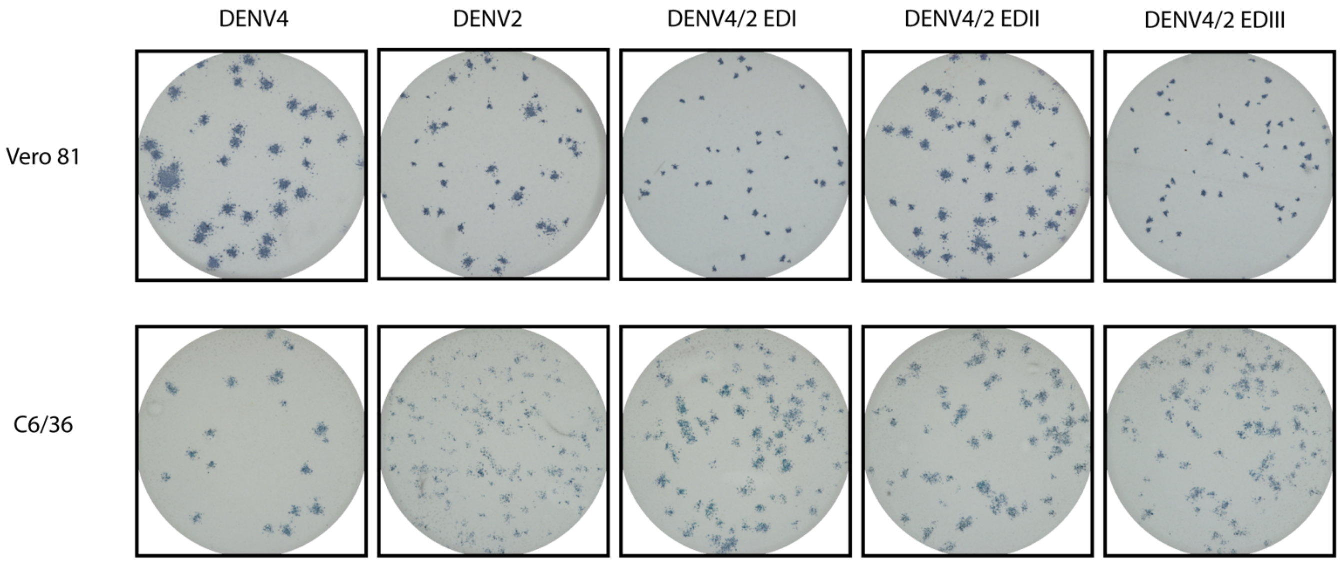

Supplemental Figure 2. Foci of viruses on Vero 81 and C6/36 cells after 48 hours incubation at 37°C and 32°C, respectively.

Supplemental Table 1. Genome copy number per focus-forming unit of mature and immature stocks with RNAse treatment.

Sample	Cell Culture	Genome copies/mL	FFU/mL	Genome copies/titer
DENV4/2-EDI	Vero81	3.45E+10	1.53E+06	2.25E+04
	Vero-furin	7.50E+09	7.50E+05	1.00E+04
DENV4/2-EDII	Vero81	4.68E+09	1.93E+06	2.42E+03
	Vero-furin	8.70E+07	1.57E+05	5.54E+02
DENV4/2-EDIII	Vero81	3.01E+09	1.17E+05	2.57E+04
	Vero-furin	1.13E+09	4.80E+05	2.35E+03

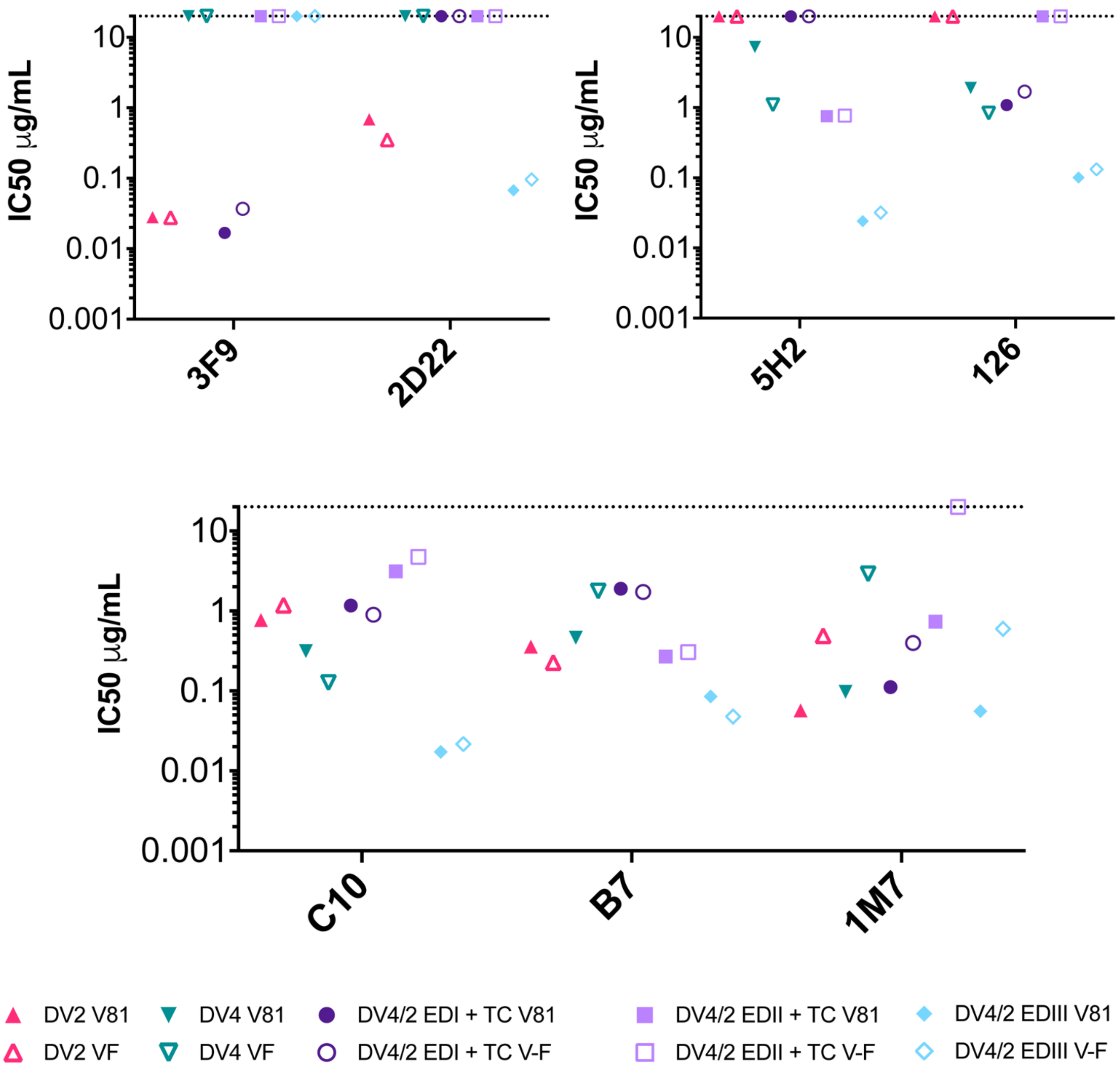

Supplemental Figure 3. FRNT of DENV4/2 ED panel comparing neutralization of partially mature (solid shapes) and mature stocks (empty shapes) by **A)** DENV2 (3F9 and 2D22) **B)** DENV4 (5H2 and 126), **C)** cross-reactive (C10, B7, and 1M7), DENV1 (1F4) and DENV3 (5J7) antibodies.

Supplemental Table 2. Origin and infection history of convalescent samples

Sample Number	Infection History	Year of Infection	Location	Time of collection
DT156	DENV1 natural infection	1983; 1997	Sri Lanka; Northern India	>15 years after first infection
DT145	DENV1 natural infection	2006	Ecuador	8 years after infection
DT147	DENV1 natural infection	2012	Trinidad, Bolivia	2 years after infection
DT001	DENV2 natural infection	2009	Sri Lanka	9 years after infection
DT110	DENV2 natural infection	1998	Kuala Lumpur	10 years after infection
DT242	DENV2 natural infection	2014	Brazil	>2 years
465	DENV2 natural infection	2019	SE Asia	>1 year
1207	DENV2 natural infection	2019	SE Asia	>1 year
1707	DENV2 natural infection	2019	SE Asia	>1 year
1737	DENV2 natural infection	2019	SE Asia	>1 year
1892	DENV2 natural infection	2019	SE Asia	>1 year
1996	DENV2 natural infection	2019	SE Asia	>1 year
2199	DENV2 natural infection	2019	SE Asia	>1 year
2396	DENV2 natural infection	2019	SE Asia	>1 year
2599	DENV2 natural infection	2019	SE Asia	>1 year
2744	DENV2 natural infection	2019	SE Asia	>1 year
002	DENV2 Tonga'74 rDEN2Δ30 Challenge	-	-	180 days after infection
006	DENV2 Tonga'74 rDEN2Δ30 Challenge	-	-	180 days after infection
007	DENV2 Tonga'74 rDEN2Δ30 Challenge	-	-	180 days after infection
008	DENV2 Tonga'74 rDEN2Δ30 Challenge	-	-	180 days after infection
009	DENV2 Tonga'74 rDEN2Δ30 Challenge	-	-	180 days after infection
010	DENV2 Tonga'74 rDEN2Δ30 Challenge	-	-	180 days after infection
011	DENV2 Tonga'74 rDEN2Δ30 Challenge	-	-	180 days after infection
013	DENV2 Tonga'74 rDEN2Δ30 Challenge	-	-	180 days after infection

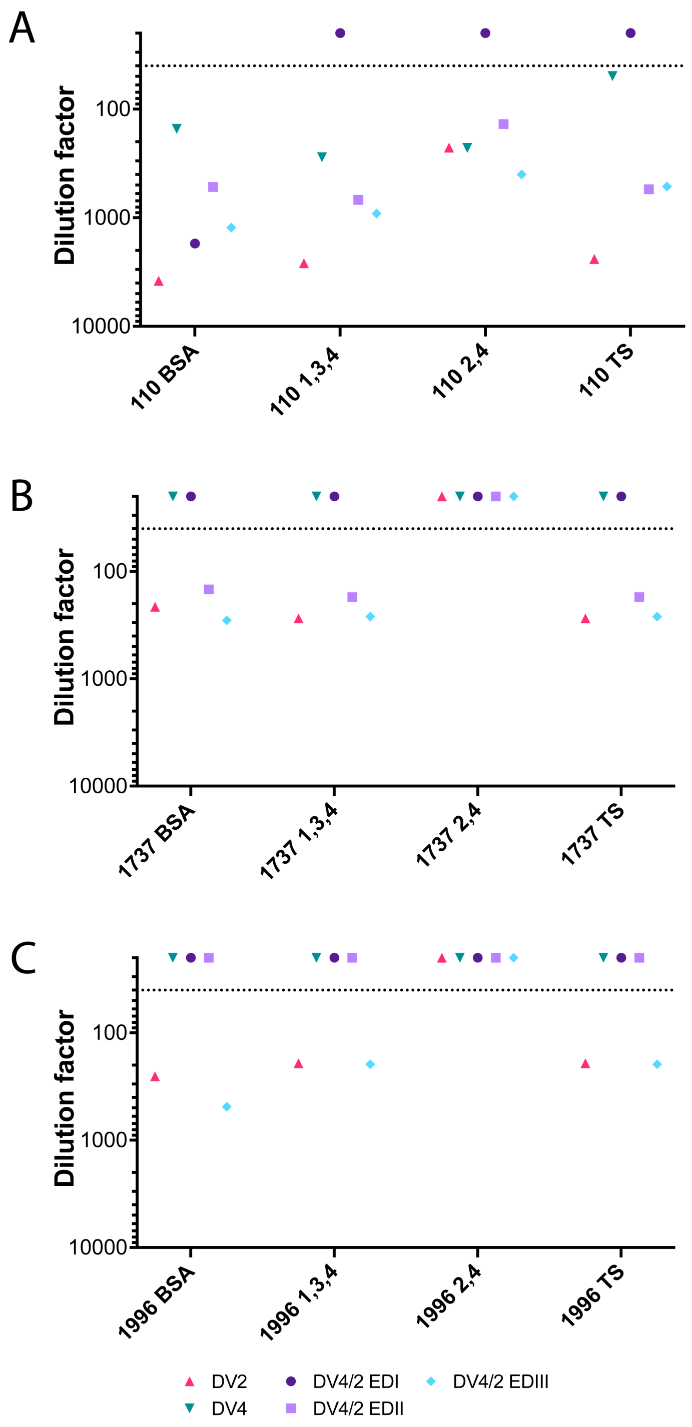

Supplemental Figure 4. FRNT of DENV4/2 ED chimeric viruses by three convalescent DENV2 samples, Panel A. 110, Panel B. 1737, Panel C. 1996 depleted by BSA control, heterotypic condition DENV1, DENV3, and DENV4, and homotypic condition DENV2 and DENV4. The type-specific (TS) antibody response is the difference between the IC50 of the heterotypic and homotypic conditions. Samples that did not neutralize were given the value of 10, below the limit of detection, 40.

Supplemental Table 3. Calculation of TS response from depleted primary DENV2 natural infection sera. “-“ indicates sample did not neutralize the virus

Sample	Virus	IC_{50} Control Depletion	IC_{50} Heterologous Depletion	IC_{50} Homologous Depletion	IC_{50} TS response*	% TS⁺
DT110	DENV2	4481.5	33560	412.8	2943.2	65.7%
1737		213	273.5	-	273.5	100%
1996		254	192.3	-	192.3	75.7%
DT110	DENV4	263.9	513.9	414.7	99.2	37.6%
1737		-	-	-	-	0%
1996		-	-	-	-	0%
DT110	DENV4/2-EDI	3420.4	-	-	-	0%
1737		-	-	-	-	0%
1996		-	-	-	-	0%
DT110	DENV4/2-EDII	1003.0	1033.8	234.7	799.1	79.7%
1737		146.8	173.2	-	173.2	100%
1996		-	-	-	-	0%
DT110	DENV4/2-EDIII	1611.0	1454.4	730.2	724.2	45.0%
1737		284.6	262.8	-	262.8	100%
1996		488.6	196.1	-	196.1	40.1%

*TS response is calculated as $IC_{50}^{heterologous} - IC_{50}^{homologous}$

⁺ Percent TS response is calculated as $\frac{IC_{50}^{heterologous} - IC_{50}^{homologous}}{IC_{50}^{control}} \times 100$

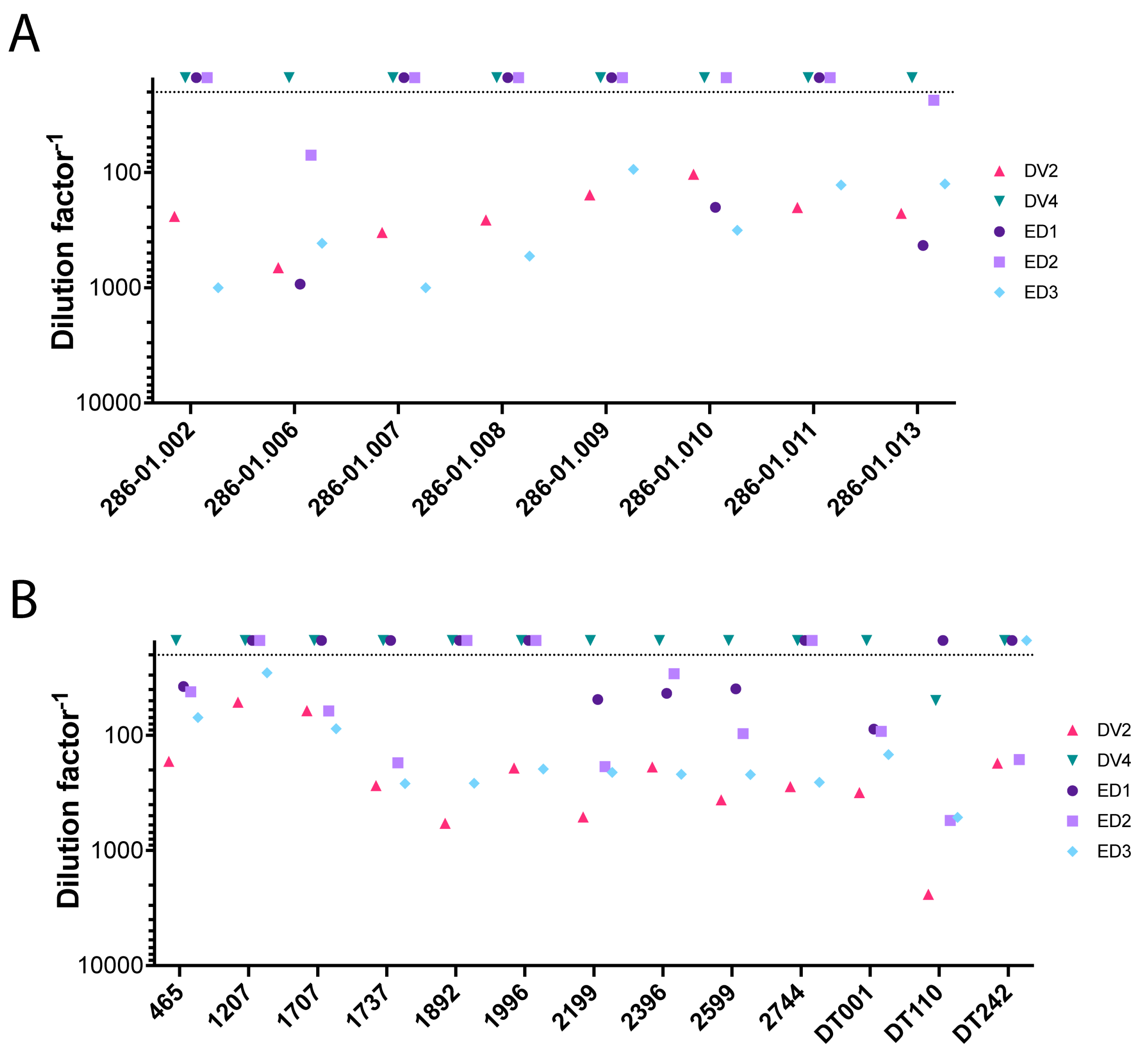

Supplemental Figure 5. FRNT of ED panel by convalescent primary DENV2 sera after A) human challenge and B) natural infection.

Supplemental Table 5. Number of individuals from human challenge, natural infection, or all cohorts targeting each envelope domain (ED) and targeting one, two, or three EDs. For example, a sample that neutralized EDII and EDIII is included under EDII, EDIII, and two targets.

**Sample**		**Domain**			**Total number of targets**		
Cohort	N	EDI	EDII	EDIII	One target	Two targets	Three targets
Human Challenge	8	3	2	8	5	1	2
Natural Infection	13	5	9	12	5	3	5
Total	21	8	11	20	10	4	7

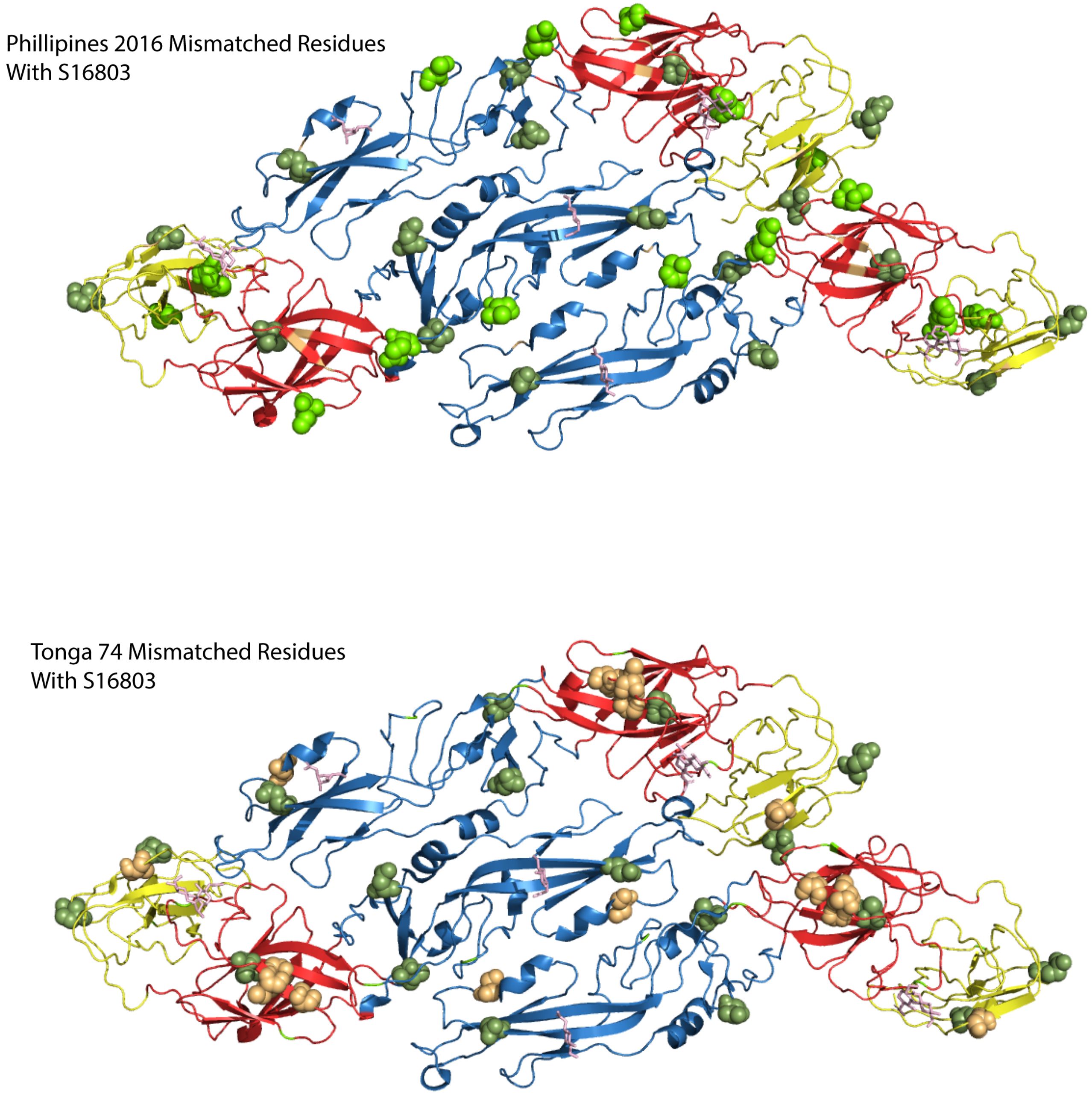

Supplemental Figure 6. Mismatched residues on E between human challenge strain Tonga ’74 and representative strain from Southeast Asia. The dark green residues are shared mismatches in both strains. The light green residues are mismatches between S16803 and Phillipines 2016. The beige residues are mismatches between S16803 and Tonga’74.

Supplemental Table 5. All primers used in this study.

Primer name	Sequence 5’-3’
DENV4 RT NS1R	GCCGTCACCTGTGATTTGAC
DENV2 RT NS1R	CCACATCAGATTCTCCAGTC
DENV4 Sanger F	CGAGAGAAACCGCGTATCAACC
DENV4 Sanger R	CGAGAGAAACCGCGTATCAACC
DENV2 Sanger F	GAGACGCAGATCTGCAGGC
DENV2 Sanger R	TCTGTGATGAAAATCCCACTGCC
DENV4 qPCR F	GTTAGCAGTTTCACCACCCC
DENV4 qPCR R	AACCTGGGCGTATCATGGAA
DENV4 qPCR Probe	56-FAM/ATGAAGCTC/ZEN/CTTCGACAGGC/3IABkFQ
DENV2 qPCR F	CACATGTGGAACGAGTGC
DENV2 qPCR R	TGGGTAACTTATGGGACTTGTA
DENV2 qPCR Probe	56-FAM/ACCACGGGA/ZEN/GAACATAGAAGAGAA/3IABkFQ
